# Marine-derived PUFAs and peptides for aging-related memory deficits: processing methods, pharmacokinetics, mechanistic insights, and clinical application

**DOI:** 10.3389/fnut.2026.1844290

**Published:** 2026-05-14

**Authors:** Zhiyou Yang, Wendi Deng, Qidong Lu, Zhengyuan Zhou, Cai Song, Syeda Noor-ul-Ain Naqvi

**Affiliations:** 1 Guangdong Provincial Key Laboratory of Aquatic Product Processing and Safety, Guangdong Province Engineering Laboratory for Marine Biological Products, Zhanjiang Municipal Key Laboratory of Marine Drugs and Nutrition for Brain Health, College of Food Science and Technology, Shenzhen Institute of Guangdong Ocean University, Guangdong Ocean University, Zhanjiang, China; 2 Mom’s Garden Institute of Nutrition and Health, Saarbrücken, Germany; 3 Inne Institute of Nutrition and Health, Saarbrücken, Germany; 4 National Institute of Food Science and Technology, University of Agriculture, Faisalabad, Pakistan

**Keywords:** cognitive function, docosahexaenoic acid, eicosapentaenoic acid, memory enhancement, peptides, PUFAs

## Abstract

Cognitive decline and memory disorders are increasingly prevalent globally, especially in aging populations, imposing substantial social, emotional, and medical burdens on individuals and healthcare systems. Food-derived dietary interventions play a critical role in the prevention and management of these conditions, with marine-derived polyunsaturated fatty acids (PUFAs) and bioactive peptides emerging as promising candidates for enhancing brain health and cognitive function. This review summarizes advanced processing techniques for these bioactive substances, including physicochemical methods for the extraction and purification of PUFAs, as well as enzyme-mediated degradation of marine proteins for peptide production. It also covers their multifaceted mechanisms underlying memory enhancement, such as antioxidant, anti-apoptotic, anti-inflammatory, and cholinergic modulation, supported by preclinical animal studies and preliminary human clinical trials. Finally, existing challenges such as low bioavailability and unstandardized formulations, along with prospects including sustainable production, personalized bioactive blends, and precision nutrition, are discussed. This review first clarifies the complementary neuroprotective mechanisms of marine-derived PUFAs and peptides, and advocates for systematic exploration to translate preclinical findings into clinical applications.

## 1 Introduction

Neurological disorders represent the leading cause of disability and the second leading cause of death worldwide. Over the past three decades, the absolute number of deaths and disabilities attributable to neurological diseases has risen substantially, especially in low- and middle-income countries (1). China’s population aged 65 years and older is projected to increase rapidly in size from 172 million in 2020 to 366 million by 2050, accounting for 40% of the global growth in this age group (2). Aging is a major risk factor for cognitive impairment, including learning and memory deficits, which are core features of dementia in the elderly. The cortical-hippocampal neural circuits are important for memory coding, consolidation, and retrieval. A study involving rhesus monkeys demonstrated that following sensory stimulus input, the prefrontal cortex regulates working memory and attention (3). The interaction between this prefrontal regulation and hippocampal persistent activity is coordinated via theta–gamma phase–amplitude coupling, thereby influencing temporal memory performance (4). Recently acquired memories are reactivated in the hippocampus via sharp-wave ripples during pupil-contracted substates of non-rapid eye movement sleep, representing an initial step in long-term memory consolidation (5). However, hippocampal-cortical circuit and sleep/wake cycle were dramatically disrupted in early phase of Alzheimer’s disease (AD) (6).

AD, a progressive neurodegenerative disorder, has become a major public health concern, severely compromising the health and quality of life of elderly individuals. Worldwide, the number of people living with dementia is projected to increase from 57.4 million (95% uncertainty interval 50.4–65.1) in 2019 to 152.8 million (130.8–175.9) by 2050 (7). Inadequate post-diagnostic support has been widely reported, affecting 37% of dementia patients in high-income countries and 45% in low- and middle-income countries. Meanwhile, 66% of patients required individualized care plans, and 37% of professional caregivers as well as 54% of informal caregivers reported persistent or frequent stress (8). Current pharmacotherapies for AD remain palliative rather than curative, providing only symptomatic relief. Accordingly, dietary interventions have emerged as a promising and sustainable strategy to alleviate AD-associated learning and memory deficits. Clinical evidence supports the cognitive benefits and neuroprotective potential of dietary patterns including the Mediterranean diet, ketogenic diet, and MIND diet (9).

This review focuses on fatty acids and bioactive peptides as key nutritional interventions exemplified by the DASH diet, ketogenic diet, and Mediterranean diet, which are rich in high-quality proteins and PUFAs. As reported by Monti et al. and Pei et al., the hippocampus exhibits high plasticity and can adapt its structure and function in response to diverse environmental and lifestyle factors, including dietary intake (10–12). Omega-3 fatty acid, especially docosahexaenoic acid (DHA), intake is associated with a reduced risk of AD (13). In elderly individuals with mild cognitive impairment (MCI), higher intakes of DHA and eicosapentaenoic acid (EPA) were associated with improved cognitive performance (14). Regular supplementation with fish oil has been shown to enhance cognitive processing and attention, accompanied by altered brain activity during working memory and long-term memory tasks (15). However, clinical outcomes of n-3 PUFAs in AD and cognitive disorders are highly heterogeneous. Key factors contributing to such heterogeneity include raw material sources, extraction methods, oxidative stability, and refining procedures, all of which further affect the purity, bioavailability, and biological activity of EPA and DHA (16).

In recent years, marine-derived peptides have also attracted increasing attention for their potential to improve cognitive function, particularly learning and memory. Major marine sources of bioactive proteins and peptides include fish, algae, mollusks, crustaceans, microorganisms, invertebrates, and marine by-products such as skin, bones, and viscera (17–20). Advances in extraction and purification technologies, including enzymatic hydrolysis, ultrafiltration, ion-exchange chromatography, high-performance liquid chromatography (HPLC), and molecular docking, have enabled efficient isolation and preparation of bioactive peptides with high purity and activity (17). Nevertheless, reported bioactivities remain inconsistent across studies. Variations in processing conditions, such as peptide aggregation, oxidation, and structural denaturation, can compromise functional efficacy (17). These methodological discrepancies complicate cross-study comparisons and highlight the urgent need for standardized optimization. Against this background, the present review aims to critically evaluate the current challenges and provide a comprehensive overview of marine-derived bioactive PUFAs and peptides, with the goal of addressing key limitations in this rapidly evolving field.

## 2 Marine-derived PUFAs: sources, processing, purification and stabilization

Long-chain PUFAs can be biosynthesized by diverse marine microorganisms, including bacteria, yeast, microalgae, protists, fungi, and dinoflagellates (21). DHA and EPA, the two most physiologically important long-chain n-3 PUFAs in humans, are almost exclusively derived from microalgae (22). Two distinct pathways are involved in the biosynthesis of these PUFAs: the anaerobic PUFA synthase pathway, which is mainly distributed in marine bacteria and eukaryotic microalgae, and the conventional aerobic fatty acid synthase (FAS) pathway in microalgae (21). The primary sources of DHA include heterotrophic microalgae, marine fish oil, and genetically engineered microbes. Specifically, the heterotrophic stramenopiles *Schizochytrium* sp. and *Aurantiochytrium* sp. are the most prolific natural and industrial producers of DHA (23, 24). As non-photosynthetic protists, these microorganisms can accumulate total fatty acids range from 23.9 to 54.5 g/L, with DHA comprising 13.2–37.9% of total fatty acids and negligible EPA content (25). Marine fish oil, derived from fatty fish such as salmon, tuna, and sardines, is a traditional dietary source of DHA. However, it is crucial to emphasize that fish cannot synthesize DHA *de novo*, they merely accumulate PUFAs through the marine food chain by ingesting DHA-producing microalgae (26). Genetically engineered yeast have also emerged as advanced sustainable sources of DHA (27). In contrast, EPA is predominantly sourced from photoautotrophic marine microalgae, marine fish and krill oil, and metabolically engineered yeast. *Phaeodactylum tricornutum* and *Nannochloropsis oceanica*, which are natural high-EPA producers that synthesize EPA via the aerobic FAS pathway with a production rate of 5.0 and 4.3% of dry weight, respectively (26, 28). Similar to DHA, fish and krill do not possess the ability to synthesize EPA *de novo*. Furthermore, the oleaginous yeast *Yarrowia lipolytica* has been successfully metabolically engineered to produce high levels of EPA, with engineered strains capable of accumulating EPA up to 15% of dry cell weight, accounting for 56.6% of total fatty acids (29).

There is an increasing demand for purified forms of PUFAs, especially DHA and EPA, with intensive investigations into their health-promoting effects (30). n-3 PUFAs can be produced in various molecular forms, including triacylglycerols, free fatty acids, and alkyl esters, through physical, chemical, and enzymatic approaches (31). Conventional approaches rely heavily on organic solvents for lipid recovery. Given their regulatory limitations in food-grade applications, alternative solvent systems including petroleum ether, methanol/water, and ethanol/water have been increasingly investigated. These extraction strategies afford satisfactory oil yields, especially when applied to fat-rich fish species such as herring, tuna, sardine, and salmon. Freeze-drying significantly improves fatty acid recovery, achieving nearly 100% yield, while ethanol-soaked viscera quadruple the FA yield compared with fresh samples and provide EPA and DHA contents comparable to hot air-dried samples (32, 33). Ultrasound-assisted extraction (UAE), microwave-assisted extraction (MAE), and supercritical CO₂ fluid extraction (SFE) represent some of the most widely applied green and safe extraction technologies. Although these approaches exhibit distinct advantages over conventional methods, further optimization is still required to minimize oxidative degradation and thereby maintain the quality of the final products. Supercritical CO₂ extraction of northern shrimp by-products at 35 MPa and 40 °C yields a deep-red oil rich in n-3 PUFAs, containing 7.8 ± 0.06% EPA and 8.0 ± 0.07% DHA (34). Similarly, the viscera of *Cucumaria frondosa* can be used as a natural source of n-3 fatty acids via supercritical CO₂ extraction. Using response surface methodology, the optimal conditions were determined as 75 °C, 45 MPa, ethanol-to-feedstock mass ratio of 2:1, 20 min static extraction, and 30 min dynamic extraction. In general, SFE is widely employed for PUFA extraction at temperatures of 40–80 °C and pressures of 10–30 MPa, and is regarded as one of the most efficient and promising techniques for recovering EPA and DHA from freshwater and marine fish (32, 35). UAE and MAE are also widely used to improve lipid and n-3 PUFAs recovery from algal biomass, such as the DHA-rich dinoflagellate *Crypthecodinium cohnii* (36, 37). Enzyme-assisted oil extraction represents an eco-friendly approach to convert underutilized fish species and processing by-products into high-quality fish oil suitable for human consumption. For instance, three commercial proteolytic enzymes (Alcalase®, Neutrase®, Protamex®) and two incubation durations (35 and 70 min) have been used to extract oil from whole Baltic herring and filleting by-products (38). High-pressure processing (HPP) can enhance the efficiency of enzymatic hydrolysis, representing an effective strategy for extracting value-added compounds (39). Ionic liquids (ILs) are used as tunable green solvents for lipid extraction from algae due to their high thermal stability and negligible volatility. They consist of organic cations and anions, remaining liquid below 100 °C, which helps reduce evaporation and enhance the solubility of n-3 PUFAs (40, 41). Deep eutectic solvents (DESs), typically formed by combining hydrogen-bond acceptors and donors, are biodegradable and more cost-effective than ILs, enabling sustainable lipid extraction from algal biomass (42). Table 1 shows the widely used processing methods of PUFAs and corresponding yields in marine-derived sources.

**Table 1:** Marine sources, production methods, yields, and applications of DHA and EPA.

Compounds	Bio-based sources	Processing methods	Typical yields	Key applications	References
DHA	Marine microalgae (*Schizochytrium* sp.)	Heterotrophic fermentation in bioreactors followed by biomass harvesting and lipid extraction using solvent extraction, UAE, MAE, or SFE	*Schizochytrium marinum* and *S. limacinum* achieved high biomass densities (~9 g/L) with stable DHA productivity of ~0.1 g/L/day under optimized conditions	Nutraceuticals; functional foods; infant formula; cognitive and visual development	(23)
	Marine microalgae (*Aurantiochytrium* sp.)	Heterotrophic fermentation using low-cost substrates followed by lipid extraction and purification	Novel *Aurantiochytrium* sp. 6–2 produced 15.8 ± 3.4 g/L DHA within 54.8 ± 12.1 g/L total fatty acids in fermented soybean medium	Sustainable DHA supply; aquaculture feed; nutraceuticals	(24)
	Marine fish oil (salmon, sardine, mackerel)	Conventional oil extraction, refining, molecular distillation, and concentration processes	Typical fish oils contain ~10–20% DHA and ~15–30% EPA depending on species	Dietary supplements; clinical nutrition	(25)
	Genetically engineered yeast (*Yarrowia lipolytica*)	Metabolic engineering combined with fermentation and downstream lipid recovery	Engineered *Yarrowia lipolytica* expressing *Δ*-4, Δ-5, Δ-6, Δ-7 desaturase and C18-22 elongase, with DHA production of 5.6% of total fatty acids	Sustainable n-3 production for food and aquaculture	(27)
EPA	Marine microalgae (*Phaeodactylum tricornutum*, *Nannochloropsis*)	Phototrophic or mixotrophic cultivation followed by solvent extraction, UAE, MAE, or SFE	*P. tricornutum* cultivated on lignocellulosic hydrolysates produced ~256 mg/L EPA with productivity of ~19.7 mg/L/day	Cardioprotective and anti-inflammatory supplements; aquafeed ingredients	(28, 29)
	Fish and krill oil	Mechanical extraction followed by purification and concentration (distillation, enzymatic hydrolysis)	Species-dependent EPA levels typically ranging 15–30% of total fatty acids	Human nutrition; treatment of hypertriglyceridemia; nutraceutical formulations	(26)
	Engineered *Yarrowia lipolytica*	Metabolic engineering and industrial fermentation with lipid recovery	Engineered strains produce EPA up to ~15% of dry cell weight, with ~56% EPA composition in total lipids	Sustainable production of n-3 oils for food and pharmaceutical industries	(29)

Purification techniques are widely applied to concentrate n-3 PUFAs (especially EPA and DHA) from marine biomass and oils, aiming to eliminate contaminants and unwanted components. These methods facilitate the conversion of natural triglycerides into free fatty acids (FFAs) or ethyl esters, thereby enabling efficient enrichment and high-purity preparation (43). Urea complexation (UC) has been used to optimize the concentration of DHA and EPA from tuna oil. Under conditions of a urea-to-fatty acid molar ratio of 15:1, crystallization at −5 °C for 20 h, the total content of DHA + EPA reached 85.02% with a liquid recovery yield of 25.1% (44). In another study, UC and molecular distillation (MD) were combined to purify EPA and DHA from sardine oil ethyl esters (SOEE). Under optimal UC conditions (urea-to-SOEE ratio 1.9:1, crystallization at −1 °C), the purity and total recovery of EPA and DHA were 65.6 and 46.8%, respectively (45). In addition to traditional refining processes, several advanced technologies have been developed for fish oil processing. As reported by Lin et al. and Espinosa et al., the optimal MD parameters were 75 °C distillation temperature, 54.8 °C preheating temperature, 4.5 °C condensation temperature, and 307 rpm rotation speed, achieving EPA + DHA purity of 83.5% (45, 46). A novel and scalable method, alkali-assisted solvent fractionation via anti-solvent precipitation, has been established for purifying PUFAs from marine thraustochytrids. The total PUFA proportion in the original biomass is 51 ± 2%. When applied to freeze-dried biomass, this method achieved 70 ± 1.5% purity and approximately 72–74% PUFA recovery. For wet biomass, PUFA recovery of approximately 33 ± 1% and purity of 77–84% were obtained (47, 48).

**Figure 1:**
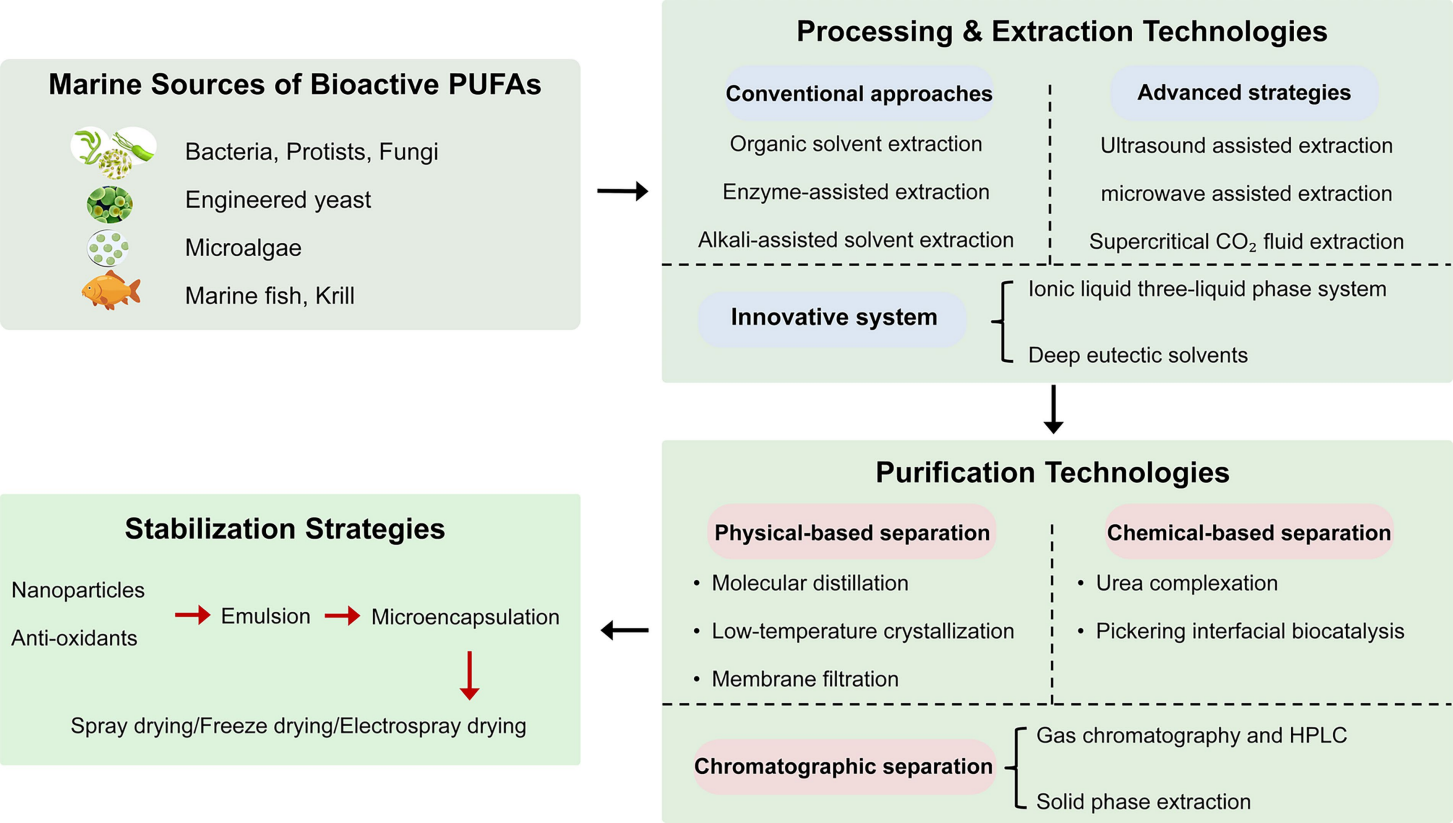
The extraction, purification, and stabilization strategies of marine-derived PUFAs.

Encapsulation and stabilization technologies are employed to suppress oxidation, improve water solubility, enhance chemical stability, and promote the bioavailability of n-3 fatty acids (49). Due to their high nutritional value and low stability against environmental and processing stresses, microencapsulation of n-3 fatty acids with antioxidants has gained increasing interest to improve stability (50). In addition, various colloidal delivery systems, including emulsions and nanoemulsions, have been successfully used to encapsulate and protect n-3-rich marine oils, especially for mixing and homogenization processes (30, 51). Fish oil-loaded capsules have been prepared via monoaxial/coaxial electrospraying and spray-drying using low-molecular-weight carbohydrates as wall materials to investigate the effects of encapsulation techniques on oxidative stability (52). Spray-drying effectively maintains long-term storage stability of oils. Compared with electrospraying, spray-drying generally produces capsules with higher encapsulation efficiency, whereas electrosprayed particles show lower retention properties (52). The extraction, purification, and stabilization strategies of PUFAs were summarized in Figure 1.

## 3 Marine bioactive peptides: sources, preparation, purification and structural identification

Marine organisms represent a rich reservoir of structurally diverse bioactive compounds. Among these, marine-derived peptides exhibit promising therapeutic potential and can be utilized as antihypertensive, antioxidative, anticoagulant, antibacterial, and neuroprotective agents in functional foods, nutraceuticals, and pharmaceutical formulations (53). Generally, the bioactivity of peptides is predominantly determined by their amino acid composition and sequence, which typically include 3–20 amino acid residues (54). Fish represent an excellent source of high-quality protein, with protein contents of approximately 17–22% in muscle tissue and 8–35% in other body parts (55). Fish by-products, including skin, bones, skulls, and viscera, are particularly abundant in proteins and bioactive nitrogenous compounds, which can be converted into bioactive peptides via enzymatic hydrolysis or microbial fermentation (56, 57). In addition to fish, marine proteins and peptides can be obtained from seaweeds, shellfish, and various seafood processing discards (58). Shellfish tissues generally contain 10–23% (w/w) high-quality protein, representing a promising resource for the discovery of biofunctional peptides (59). Marine macroalgae and microalgae constitute a heterogeneous group of organisms with diverse health-promoting bioactivities, and have recently been recognized as sustainable sources of biofunctional proteins and peptides (60, 61). Other important marine protein sources include molluscs (mussels, oysters, sea cucumbers), cephalopods (squid and octopus), and finfish (salmon, tuna and cod) (62). Aquaculture now supplies more than half of global seafood consumption and represents an expanding source of marine proteins. These proteins are nutritionally valuable due to their well-balanced essential amino acid profiles (63). The marine-derived memory enhancing peptides are summarized in Table 2. The commercial translation of marine-derived proteins and peptides remains hindered by challenges related to large-scale production and stable raw material supply. A deeper understanding of their physiological functions, molecular mechanisms of action, and clinical efficacy will facilitate their application as functional ingredients in foods, cosmetics, and biomedical products (58).

**Table 2:** Marine-derived bioactive peptides targeting memory enhancement: sources, enzymolysis conditions, peptide sequences, and mechanisms.

Marine Sources	Enzymolysis conditions (enzymes used)	Peptide sequence	Dominant amino acids	Mechanisms	References
Tilapia head	Protease hydrolysis (alkaline protease, papain)	Peptides with molecular weights < 3 kDa	Glu, Gly, Pro	Improves cognitive performance; antioxidant defense	(131)
*Aspergillus flavus*	—	Cyclo-(L-Pro-L-Phe)	Pro, Phe	Neuronal protection	(109)
Chum salmon skin	Protease hydrolysis (papain, trypsin, alkaline protease)	MCPs (GP-Hyp peptides)	Gly, Pro, Hyp	Antioxidant activity; increases CREB phosphorylation and brain-derived neurotrophic factor (BDNF) expression	(102)
Lantern fish (*Benthosema pterotum*)	Protease hydrolysis (Protease N)	FYY; DW	Phe, Tyr, Trp, Asp	Reduces ROS; anti-apoptotic; increases BDNF and antioxidant enzymes	(103)
Indian squid (*Loligo duvauceli*)	Protease hydrolysis (α-chymotrypsin, trypsin, pepsin)	WCTSVS	Trp, Cys, Ser	Antioxidant activity; prevents DNA damage; inhibits lipid peroxidation	(171)
Anchovy (*Coilia mystus*)	Protease hydrolysis (Alcalase, papain, pancreatin)	PAYCS; CVGSY	Pro, Ala, Tyr, Cys	AChE inhibition; antioxidation; reduces ROS and Ca^2+^ influx	(100, 101)
Sea cucumber (*Holothuria scabra*)	Protease hydrolysis (papain)	CFH (hydrolysates); SFGDI	Ser, Phe, Gly, Asp	Antioxidant activity; enhances neuronal survival and synaptic plasticity	(106, 107)
Shrimp	Alcalase® Food Grade	QMDDQ; KMDDQ	Met, Asp., Gln	Inhibits neuronal apoptosis; activates CREB-BDNF signaling	(172)
Antarctic krill	Protease hydrolysis (trypsin)	SSDAFFPFR; SNVFDMF; FPF	Ser, Asp., Phe, Arg	Memory enhancement; increases CREB, SYN, PSD-95; reduces AChE	(104, 105)
Oyster	Protease hydrolysis (neutral protease or compound proteinase)	OPH; OPU	His, Pro, Tyr	Reduces AChE; increases ChAT and BDNF; anti-inflammatory effects	(98, 99)
Seahorse	Protease hydrolysis (trypsin, α-chymotrypsin, papain, and pronase E)	HTP-1 (GTEDELDK)	Glu, Asp., Lys	Protects neurons from amyloid β (Aβ)-induced toxicity; increases Bcl-2 expression	(97)

Several strategies have been established for the preparation of marine-derived bioactive peptides, including solvent extraction, chemical hydrolysis, enzymatic hydrolysis, microbial fermentation, and simulated gastrointestinal digestion. Among these approaches, enzymatic hydrolysis serves as the predominant technique for extracting bioactive peptides from marine biomass, as it is generally recognized as safe process. This mild and controllable method not only preserves peptide structural integrity and bioactivity but also improves product stability and ensures good experimental reproducibility (64). In addition, green solvent systems, particularly deep eutectic solvents, have exhibited excellent extraction performance, achieving peptide recovery rates of 96–100% from marine collagen sources (64, 65). Microbial fermentation also represents a sustainable biotechnological route for peptide production. Fermentative proteolysis can generate novel cyclic and functional peptides with enhanced biological activities (17). A variety of proteases for enzymatic hydrolysis are commonly employed, including plant-derived enzymes (pepsin, papain, bromelain), animal-derived enzymes (pancreatin, trypsin), and microbial enzymes (neutrase, flavourzyme, alcalase) (66). The efficiency of enzymatic hydrolysis is significantly affected by enzyme type, enzyme-to-substrate ratio, temperature, pH, and hydrolysis time. Optimization of these parameters is essential to improve peptide yield and biological activity. Recent studies have indicated that controlled and selective enzymatic hydrolysis can enhance the functional properties of peptides, including gelation, emulsification, and oxidative stability (67). Comparative hydrolysis using different proteases has also been shown to improve the oxidative stability and interfacial adsorption of protein emulsions (66). Optimization studies using alcalase and flavourzyme have revealed notable improvements in hydrolysis efficiency and the antioxidant activity of derived peptides (68). Response surface methodology (RSM) and statistical modeling are powerful tools for optimizing the enzymatic hydrolysis of proteins. RSM based on central composite design has been used with alcalase and trypsin to optimize hydrolysis conditions for *Scenedesmus obliquus* proteins. Key parameters considered during optimization included enzyme concentration, hydrolysis time, reaction temperature, and pH. Under optimized conditions, the maximum degrees of hydrolysis reached 16.7% for alcalase and 15.51% for trypsin (68). Well-controlled hydrolysis facilitated the production of low-molecular-weight peptides (3–10 kDa) with superior functional properties, including high DPPH radical scavenging activity (approximately 80%), improved emulsifying capacity (up to 112 m^2^/g), enhanced fat-binding capacity, and increased foaming ability (69). These findings confirm that optimized hydrolysis systems can significantly improve the bioactivity and functional performance of microalgal peptides, supporting their potential application as neuroprotective and antioxidative agents.

Purification is a critical step for isolating peptide fractions with high bioactivity, purity, and safety. Membrane filtration techniques, especially ultrafiltration using 3, 5, and 10 kDa cutoff membranes, have been effectively used for the molecular weight-based fractionation of hydrolyzed *Scenedesmus obliquus* peptides (69). Bioactive peptides can be further purified using chromatographic methods, including ion-exchange chromatography, gel filtration, affinity chromatography, and reversed-phase HPLC. Multistep chromatographic strategies have been successfully applied to isolate antioxidant and immunomodulatory peptides from microalgal and marine hydrolysates (70). In addition, solid-phase extraction offers a rapid, cost-effective technique with high peptide recovery, making it suitable for the scalable purification of bioactive peptides while maintaining their functional efficiency (71).

**Figure 2:**
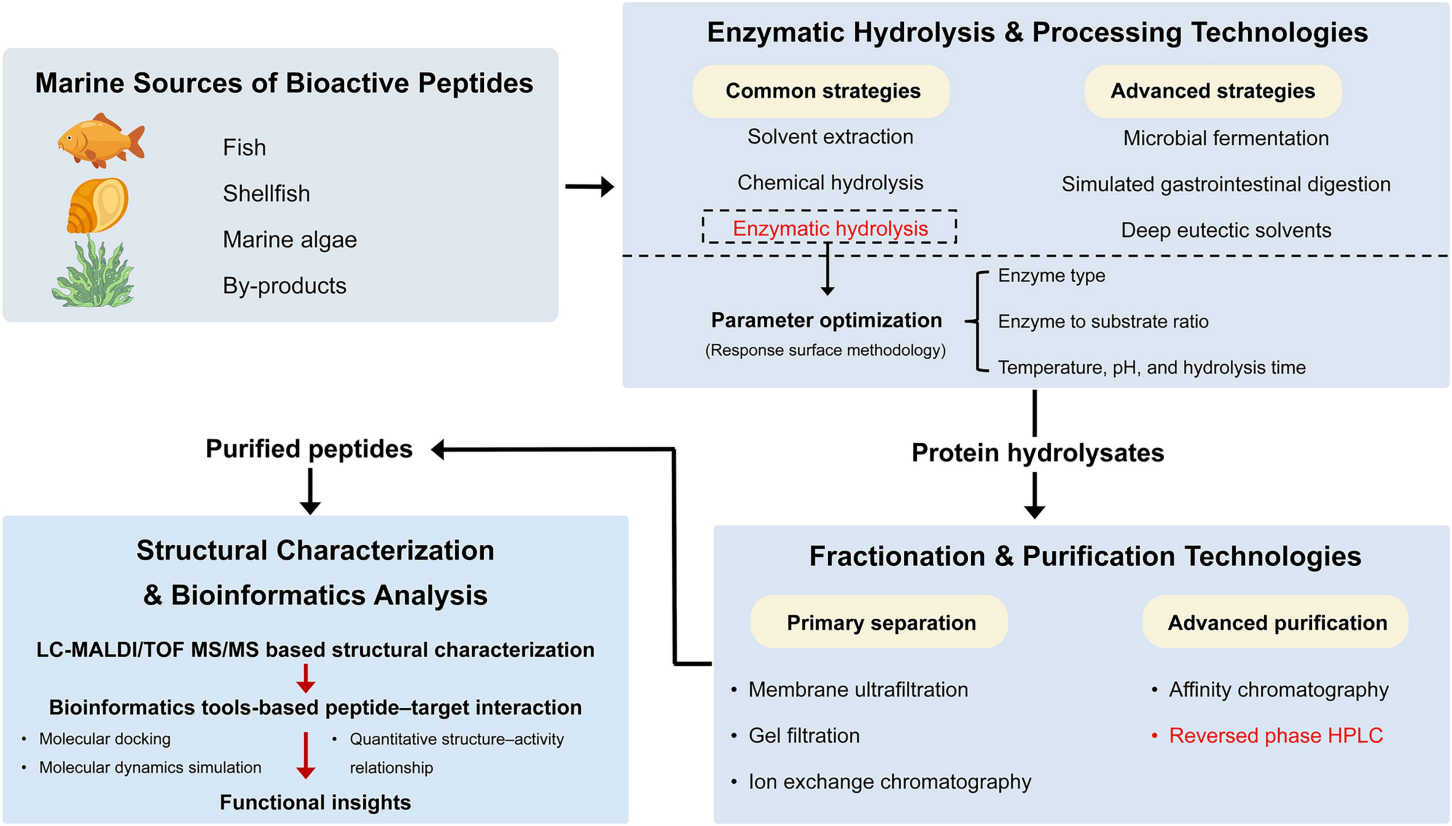
Comprehensive production pipeline of marine-derived bioactive peptides: from source to functional characterization.

Advanced mass spectrometry-based platforms are widely used for the structural identification of bioactive peptides, including matrix-assisted laser desorption/ionization time-of-flight mass spectrometry (MALDI-TOF MS), liquid chromatography–tandem mass spectrometry (LC–MS/MS), and electrospray ionization mass spectrometry (ESI-MS). These high-resolution techniques enable accurate determination of molecular mass, amino acid sequence, and structural characteristics, greatly simplifying peptide identification from complex biological matrices (72). Beyond experimental identification, computational and bioinformatics approaches have become indispensable for exploring peptide function and mechanisms of action. Molecular docking, molecular dynamics simulation, and quantitative structure–activity relationship (QSAR) modeling are increasingly used to predict peptide–target interactions, binding affinity, and structure–activity relationships (17). These in silico strategies facilitate the screening of potential bioactive compounds and the elucidation of underlying molecular mechanisms by integrating peptide structural information with biological targets and signaling pathways (73). Collectively, mass spectrometry-based peptidomics and bioinformatics modeling provide a robust platform for the identification, characterization, and functional prediction of marine-derived bioactive peptides (Figure 2).

## 4 Underlying mechanisms of marine-derived PUFAs on learning and memory

### 4.1 Preclinical and clinical evidence of PUFAs on cognitive memory

DHA is highly enriched in neuronal membranes and contributes to the maintenance of normal neurological function and neuronal homeostasis (74). In mouse models, dietary n-3 PUFA deficiency leads to sustained depletion of brain DHA content, which can trigger long-lasting impairments in cognitive function if not corrected during early life (75). Brain DHA concentrations decline significantly with advancing age and in neurodegenerative disorders such as AD, and this reduction is closely associated with deteriorated hippocampus-dependent spatial learning and memory performance (76). Adequate brain DHA levels can attenuate the detrimental effects of stress, a key risk factor for age-related cognitive decline, and modulate neuronal and astroglial activities that underpin synaptic transmission in the hippocampus (77). The cognitive benefits of n-3 PUFAs are further modulated by dietary fatty acid formulations. Reducing the dietary n-6:n-3 PUFA ratio improved cognitive performance in the Morris water maze test and upregulated the expression of peroxisome proliferator-activated receptor *α* (PPARα) and PPARγ. Animals fed the lowest n-6:n-3 PUFA ratio exhibited marked enhancements in PPAR transcriptional activity and spatial learning ability (78).

Accumulating clinical trials suggest that n-3 PUFA supplementation is associated with potential benefits for cognitive performance in human subjects across all life stages, although clinical outcomes remain heterogeneous due to differences in dosage, formulation, study population and baseline nutritional status. The European Food Safety Authority (EFSA) reported the safe level of intake of 1 g/day for supplemental DHA alone for all population groups (79). A relatively higher DHA supplementation (600 mg/day) exerts selective improvements in children (aged 7–9 years), it significantly enhances reading ability only in the subgroup with extremely poor reading performance (≤20rd centile) and broadly improves parent-rated ADHD-related behavioral symptoms (80). A randomized controlled trial involving 176 healthy adults aged 18–45 showed that only among those with low habitual DHA intake, daily supplementation with 1.16 g of DHA for six months significantly improved episodic memory in women and working memory reaction time in men (81). Meta-analysis on 24 studies (*n* = 9,660; follow-up 3 to 36 months) found that 40-aged and elderly individuals with a daily intake surpassing 500 mg of n-3 PUFA and up to 420 mg of EPA may improve executive function, especially when dietary DHA + EPA intake is not severely deficient (82). Furthermore, a systematic review of 7 randomized controlled trials demonstrated that n-3 PUFAs could improve Full-Scale IQ, information processing, and digit span/working memory/attention in subjects with MCI (83).

### 4.2 Mechanistic insights of PUFAs on cognitive memory

**Figure 3:**
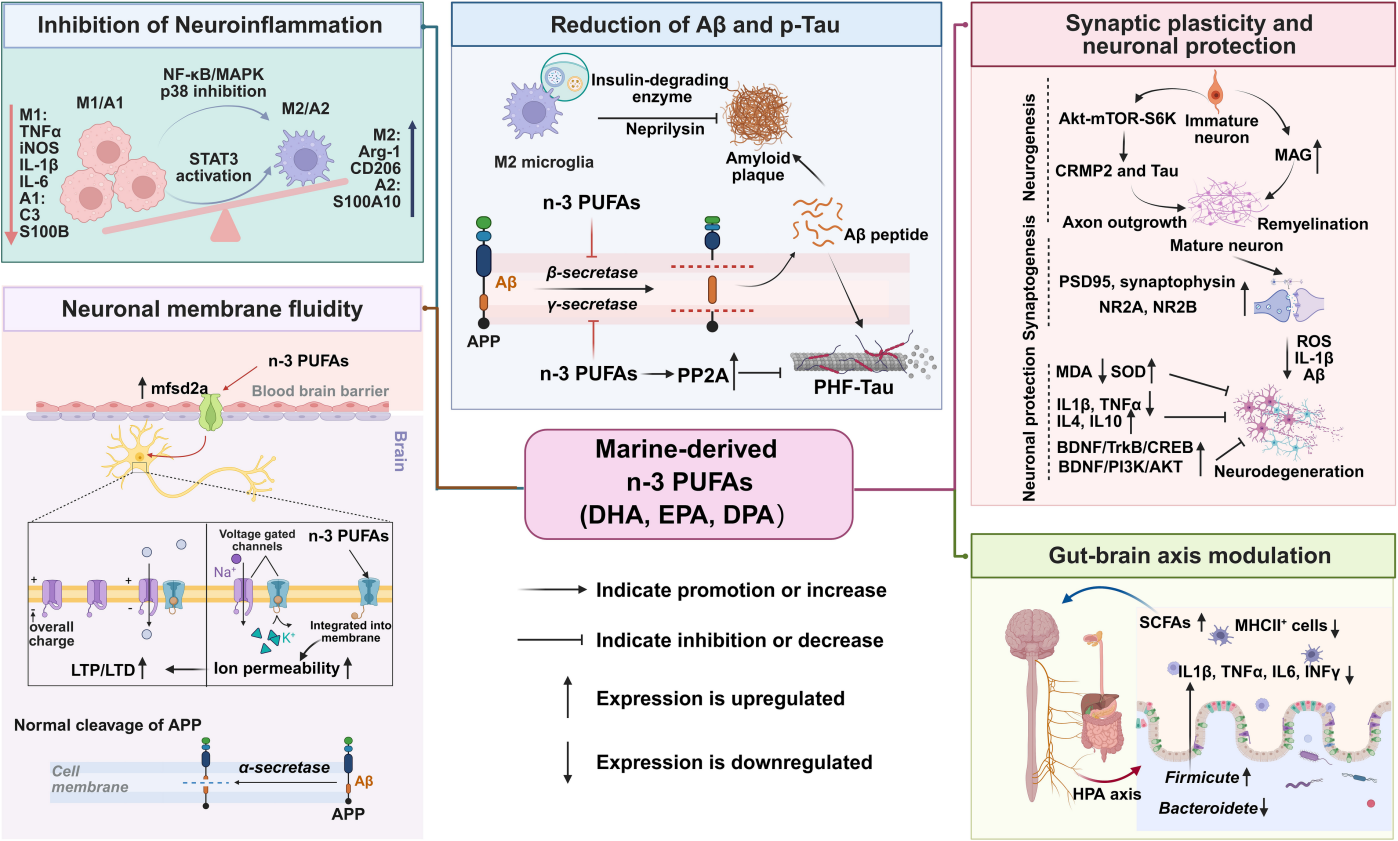
The involved signaling pathways of marine-derived PUFAs on learning and memory.

Marine-derived n-3 PUFAs, especially DHA, EPA, and DPA, are critical for preserving neuronal structure and function. These bioactive lipids serve as key components of neuronal membranes and support numerous neuroprotective processes that help ameliorate cognitive decline (16). The beneficial effects of marine-derived PUFAs are mediated through diverse biological pathways, including anti-neuroinflammatory activity, modulation of Aβ pathology, neuronal protection, regulation of the gut–brain axis, and preservation of membrane fluidity and blood–brain barrier (BBB) integrity (Figure 3).

#### 4.2.1 Inhibition of glial hyperactivation and neuroinflammation

Neuroinflammation is a major pathological driver of cognitive impairment and neurodegenerative diseases. Excessive glial activation promotes neuroinflammation and subsequent neural damage. Mechanistically, DHA and EPA inhibit microglial M1 pro-inflammatory polarization and promote a shift toward the M2 anti-inflammatory phenotype (84). Daily oral administration of 500 mg/kg DHA or EPA for 8 weeks reduced the levels of tumor necrosis factor-*α* (TNF-α), interleukin-1β (IL-1β), and IL-6 via modulating glial polarization in 24-month-old ageing rats (85). Additionally, DHA-enriched phospholipids inhibit lipopolysaccharide (LPS)-induced IL-6 synthesis and downstream signal transducer and activator of transcription 3 (STAT3) signaling in microglia, further mitigating cytokine-mediated neural damage *in vitro* and *in vivo* (86). Furthermore, DPA may protect neurons against neuroinflammation-induced injury by regulating the polarization balance between microglial M1 and M2 phenotypes, inhibiting the microglial NF-κB and MAPK p38 signaling pathways, and activating the neuronal BDNF/TrkB-PI3K/AKT signaling pathway (87).

#### 4.2.2 Reduction of amyloid-*β* plaques and tau pathology

Aβ peptide accumulation and tau protein hyperphosphorylation are core pathological hallmarks of AD, leading to neurodegeneration and synaptic dysfunction. Recent studies reveal that incorporation of DHA into neuronal membrane phospholipids alters the fluidity and composition of lipid-raft structures, thereby reducing the activity of β- and *γ*-secretases involved in the amyloidogenic processing of amyloid precursor protein (APP). This reduces Aβ production and aggregation even under conditions of n-3 PUFA deficiency (88). While n-3 PUFAs do not directly dephosphorylate tau, they enhance the activity of protein phosphatase 2A (PP2A), a major tau phosphatase, by attenuating oxidative stress and neuroinflammatory signaling cascades, thereby reducing aberrant tau phosphorylation and aggregation in neuronal models (89, 90). Additionally, the broad neuroprotective benefits associated with long-chain omega-3 intake, such as reduced apoptotic signaling and improved neurotrophic support, may promote cytoskeletal integrity and resistance to tau-mediated neurotoxicity (90). These multifaceted actions highlight the potential of marine-derived lipids to attenuate core AD pathological features and slow disease progression by targeting both Aβ- and tau-related pathways.

#### 4.2.3 Neuroprotection and enhancement of synaptic function

Marine-derived PUFAs significantly influence neuronal survival and synaptic function through neurotrophic and antioxidant mechanisms. As the most abundant n-3 PUFA in the brain, DHA is indispensable for maintaining synaptic transmission and neuronal membrane integrity (82). Studies have demonstrated that n-3 fatty acids upregulate BDNF levels, which support hippocampal neurogenesis, synaptic plasticity, and neuronal survival—processes essential for learning and memory (91). Furthermore, n-3 PUFAs protect neurons from oxidative stress and metabolic dysfunction, two pathological conditions closely associated with neurodegenerative diseases (92).

#### 4.2.4 Modulation of the gut–brain axis

The gut–brain axis (GBA) is a complex bidirectional network that integrates neural, immunological, endocrine, and metabolic signals between the gastrointestinal tract and the central nervous system. Marine-derived n-3 PUFAs, especially DHA and EPA, modulate this axis by regulating gut microbiota composition, enhancing intestinal barrier integrity, and reducing systemic and neuroinflammation (93). These PUFAs increase the abundance of beneficial microbial taxa and short-chain fatty acid-producing bacteria, which promote anti-inflammatory signaling and enhance neural resilience. Furthermore, n-3 PUFAs preserve BBB integrity and facilitate neurotransmission by modulating immune and metabolic processes centered on the gut–brain axis (94).

#### 4.2.5 Regulation of membrane fluidity and blood–brain barrier integrity

n-3 PUFAs are essential structural components of neuronal lipid membranes and play a pivotal role in maintaining membrane fluidity and signal transduction. DHA is highly enriched in neuronal membranes and regulates receptor activity, neurotransmitter release, and synaptic communication (74). Increasing the proportion of PUFAs in neuronal membranes enhances membrane fluidity and reduces pathogenic Aβ peptide formation under n-3 PUFA-deficient conditions (88). Moreover, an optimal lipid composition contributes to the maintenance of BBB integrity, which is critical for protecting the central nervous system from circulating toxins and inflammatory mediators (95).

## 5 Mechanisms of marine-derived peptides on learning and memory

### 5.1 Mechanistic insights of marine-derived bioactive peptides on cognitive memory

**Figure 4:**
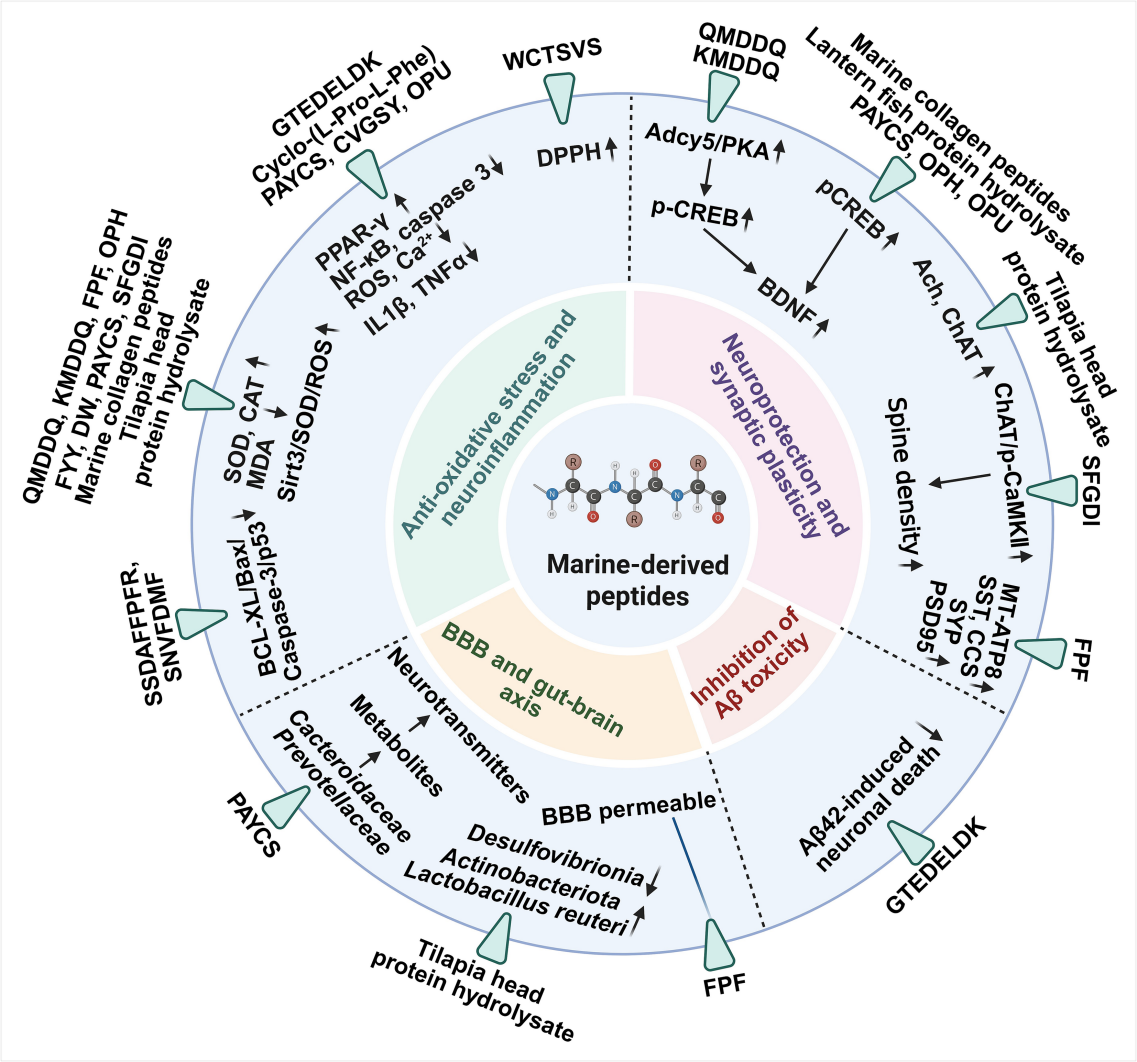
Molecular pathways of marine-derived peptides in cognitive function.

Marine-derived bioactive peptides have emerged as promising candidates for mitigating cognitive decline through multiple neuroprotective mechanisms. Evidence from preclinical studies indicates that peptides isolated from marine organisms can regulate oxidative stress, neuroinflammation, amyloid pathology, and synaptic dysfunction. Table 2 summarizes several marine-derived peptides, their enzymatic hydrolysis conditions, peptide sequences, and reported mechanisms associated with memory enhancement. Based on available experimental evidence, the cognitive protective effects of these peptides can be categorized into several mechanistic pathways (Figure 4).

#### 5.1.1 Inhibition of Aβ aggregation

Peptides derived from amphibians and marine organisms have shown anti-amyloidogenic and neuroprotective properties. The neuroregenerative peptide SLKP (Ser-Leu-Lys-Pro), derived from amphibian neuropeptides, effectively crosses the BBB to exert central effects, while inhibiting Aβ aggregation, promoting neurite outgrowth, and stabilizing microtubule structures (96). Similarly, the seahorse-derived peptide HTP-1 (GTEDELDK) protects neuronal cells from Aβ-induced cytotoxicity by increasing the production of anti-apoptotic proteins (especially Bcl-2), promoting neuronal survival and preserving neuronal integrity (97).

#### 5.1.2 Inhibition of oxidative stress and neuroinflammation

Several marine-derived peptides have demonstrated anti-neuroinflammatory effects by modulating oxidative stress and neurotransmitter imbalance. For example, oyster-derived peptides (OPH and OPU) have been reported to improve spatial learning and memory in experimental models while reducing AChE activity and increasing choline acetyltransferase (ChAT) levels, thereby restoring cholinergic neurotransmission and attenuating inflammatory responses in neural tissues (98, 99). Similarly, anchovy-derived peptides such as PAYCS and CVGSY demonstrate antioxidant activity and reduce intracellular ROS and Ca^2+^ influx, thereby protecting neuronal cells from oxidative and inflammatory damage (100, 101). These findings suggest that marine peptides may suppress glial hyperactivation and maintain neuronal homeostasis.

#### 5.1.3 Neuroprotection and enhancement of synaptic plasticity

Marine-derived peptides have shown the strong ability to enhance synaptic plasticity, which is essential for learning and memory formation. Collagen peptides derived from chum salmon skin have demonstrated antioxidant activity and increased phosphorylation of CREB and BDNF, both of which are key regulators of synaptic plasticity and neuronal survival (102). Similarly, lantern fish peptides such as FYY and DW reduce ROS generation and enhance antioxidant enzyme activity, thereby protecting neuronal cells from oxidative damage while promoting BDNF expression (103). In addition, Antarctic krill-derived peptides including SSDAFFPFR, SNVFDMF, and FPF have shown memory-enhancing properties by increasing the expression of synaptic proteins such as SYN and PSD-95, while simultaneously reducing AChE activity (104, 105). Sea cucumber peptides including SFGDI and CFH also exhibit antioxidant effects and promote neuronal survival by improving synaptic plasticity and neuronal function (106, 107). Collectively, these studies highlight the important role of marine peptides in protecting neurons against oxidative stress and supporting synaptic communication required for cognitive function.

#### 5.1.4 Blood–brain barrier penetration and integrity

Maintaining the integrity of neuronal membranes and the BBB is crucial for protecting the brain from circulating toxins, inflammatory mediators, and xenobiotic substances (108). Recent advances in humanized *in vitro* BBB microphysiological systems (BBB–MPS) have shown that collagen I microfibers (CMFs) incorporated into hydrogels accelerate brain capillary network formation, support astrocyte survival, and promote BBB maturation, highlighting the role of extracellular matrix components in preserving barrier integrity and neuronal support (108). Marine-derived peptides can interact with neuronal membranes or cross the BBB, offering neuroprotective potential. For instance, the Cyclo-(L-Pro-L-Phe) peptide has been predicted to traverse the BBB using PreADMET and showed high stability against enzymatic hydrolysis, suggesting direct neuroprotective effects within the central nervous system (CNS) (109). Similarly, krill-derived peptides such as FPF exhibit favorable digestive and absorptive properties, enabling systemic distribution and interaction with neural tissues (104, 105).

Collectively, these studies demonstrate that marine-derived peptides exert potent memory-improving and neuroprotective effects through multiple complementary pathways. They can alleviate oxidative stress and neuroinflammation, inhibit amyloid-*β* aggregation and related neurotoxicity, enhance synaptic plasticity by regulating key neurotrophic factors and synaptic proteins, as well as penetrate or preserve the integrity of the blood–brain barrier. Together, these multi-modal actions enable marine peptides to maintain neuronal homeostasis, protect against cognitive impairment, highlighting their great potential as safe and effective candidates for the prevention and intervention of neurodegenerative disorders.

### 5.2 Common and distinct neuroprotective mechanisms of marine PUFAs and peptides

Marine-derived PUFAs and bioactive peptides share several overlapping neuroprotective pathways but also exert distinct and complementary effects to counteract aging-related memory deficits. Both of PUFAs and peptides exert antioxidant, anti-neuroinflammatory, and anti-apoptotic activities, as well as the ability to upregulate the BDNF/CREB signaling pathway to enhance synaptic plasticity and neuronal survival. However, marine PUFAs predominantly maintain neuronal membrane fluidity, modulate the gut–brain axis, and preserve BBB integrity. In contrast, marine peptides mainly inhibit Aβ aggregation and regulate cholinergic system function to restore neurotransmission. Thereafter, PUFAs optimize the membrane microenvironment to facilitate peptide penetration across the BBB, while peptides may protect PUFAs from oxidation and amplify neurotrophic signaling. Through these coordinated actions, combined administration of PUFAs and peptides could generate synergistic effects to improve learning and memory.

### 5.3 Key neuroactive amino acids: tryptophan and tyrosine in cognitive regulation and neuroimmune modulation

Notably, marine-derived peptides containing aromatic amino acids have been shown to possess important neuroprotective activities. Therefore, this section focuses on the roles of tryptophan and tyrosine, two key aromatic amino acids, in cognitive memory modulation. Tryptophan (a serotonergic precursor) and tyrosine (a dopaminergic precursor) have been identified as critical modulators of mood, behavior, and cognitive function (110).

Beyond its role as a precursor for serotonin biosynthesis, tryptophan also directly regulates spatial learning and object-recognition memory (111). Acute tryptophan depletion in a double-blind, placebo-controlled study impaired long-term episodic memory and basic spatial memory, which was associated with reduced plasma tryptophan concentrations (112). In contrast, administration of a 4-week tryptophan-enriched diet to healthy older adults improved recognition of positive emotional stimuli (113). In male rats, 4-week low-dose tryptophan supplementation enhanced learning and memory, whereas high-dose tryptophan significantly impaired spatial and recognition memory; these cognitive effects were correlated with decreased serotonin levels and elevated kynurenic acid production. Notably, high-dose tryptophan administration also upregulated the expression of IL-6, IL-1*β* and TNF-*α* in the brain (114). However, acute tryptophan administration (5 and 10 mg/kg) for 3 days significantly enhanced spatial and object-recognition memory independent of serotonin levels, instead promoting microtubule polymerization by binding to α/β-tubulin heterodimers (111). Moreover, dietary supplementation with 0.5% tryptophan markedly alleviated cognitive impairment and Aβ plaque deposition in APP/PS1 mice, concomitant with upregulation of aryl hydrocarbon receptor and inhibition of the NF-κB pathway via modulation of the gut microbiota (115).

Age-related alterations in brain dopaminergic signaling contribute substantially to cognitive decline. As the primary precursor for catecholamine neurotransmitters (dopamine and norepinephrine), increased tyrosine intake favorably modulates catecholamine-dependent psychological and cognitive functions (116). Depletion of tyrosine/phenylalanine impaired stimulus processing during working memory tasks in young healthy volunteers (117). A further double-blind, randomized, placebo-controlled study demonstrated that T/T homozygotes (individuals with potentially lower striatal dopamine levels) exhibited greater cognitive benefits from tyrosine supplementation than C/C homozygotes (individuals with higher striatal dopamine levels), verifying the cognitive-enhancing role of tyrosine (118). Tyrosine has been shown to improve cognitive performance in young adults, particularly under high environmental or cognitive challenge. Furthermore, tyrosine intervention attenuates age-related cognitive decline by restoring catecholaminergic transmission (119). Key benefits of tyrosine include enhanced working memory, improved attention and concentration, and increased stress resilience. These effects are particularly pronounced under demanding conditions such as extreme environments or high cognitive load (116). Of note, 14-day daily administration of a tryptophan-tyrosine dipeptide (1 mg/kg) significantly improved spontaneous alternation in the Y-maze test in aged mice, likely by enhancing dopaminergic activity (120). Therefore, in high-intensity daytime learning conditions, tyrosine supplementation serves as an effective nutritional strategy to support dopaminergic neurotransmission in the prefrontal cortex and maintain concentration and executive function.

Collectively, these findings highlight the significant value of marine-derived tryptophan and tyrosine as promising nutraceutical or therapeutic agents for preventing memory deficits and sustaining cognitive health.

## 6 Pharmacokinetics of marine-derived PUFAs and peptides: bioavailability and dosing strategies

DHA is used as the representative example in this section due to its relatively complete pharmacokinetic data, while key pharmacokinetic features of EPA and marine bioactive peptides are also summarized.

### 6.1 Absorption, distribution, metabolism, and brain uptake of PUFAs

DHA and EPA exhibit similar absorption pathways in the gastrointestinal tract, but the brain and retina are unique in having very high levels of DHA but virtually no EPA (121). As the most abundant n-3 PUFA in the brain, DHA can penetrate the luminal membrane of brain endothelial cells, but its poor aqueous solubility impedes cytosolic transfer to the abluminal membrane, necessitating intracellular carrier proteins to facilitate its BBB translocation (122). The cellular uptake of DHA at the BBB is primarily mediated by fatty acid transport protein 1 (FATP1) and fatty acid-binding protein 5 (FABP5) (123, 124). The indispensable role of FABP5 in CNS DHA exposure is further validated by its capacity to bind DHA and mediate its uptake in brain endothelial cells and subsequent BBB transit (123). Notably, AD is accompanied by reduced cerebral DHA levels and downregulated BBB expression of FABP5, implying that upregulating FABP5 expression at the BBB may represent a novel strategy to enhance DHA brain delivery in AD. Peroxisome proliferator-activated receptor *γ* (PPARγ) can modulate FABP5 expression at the BBB, thereby facilitating DHA transport across the barrier and restoring cerebral DHA homeostasis in AD (125). EPA shows lower BBB permeability compared with DHA, and its accumulation in the CNS is more limited (121).

Emerging evidence indicates that the molecular form of DHA significantly impacts its bioavailability and brain delivery. Phospholipid- and LPC-bound DHA exhibit more efficient BBB transport via the Mfsd2a transporter compared with traditional triglyceride-based DHA supplements. Oral administration of lysophosphatidylcholine (LPC)-DHA to normal adult mice at 40 mg/kg for 30 days increased brain DHA content by > 2-fold (126). AceDoPC® (a neuroprotective structured glycerophospholipid) is a specialized DHA-based compound designed to target the brain. Compared with DHA-containing phosphatidylcholine (PC) and non-esterified DHA, AceDoPC serves as a unique and superior carrier for DHA delivery to the brain (127). Ex vivo autoradiography in rats has shown that DHA derived from AceDoPC accumulates in brain regions critical for memory, cognition, and emotional processing, confirming its efficacy in targeted cerebral DHA delivery (128). Compared with free EPA, dietary LPC-EPA could increase 100-fold brain EPA owing to the specific Mfsd2a transporter pathway (121). Following BBB crossing, DHA is esterified into membrane phospholipids and recycled among them, maintaining its distribution in brain tissues. During neurotransmission and post-brain injury, DHA is released from membrane phospholipids and converted into bioactive mediators, which regulate signaling pathways critical for synaptogenesis, neuronal survival, and neuroinflammation processes relevant to the treatment of neurological diseases (129).

### 6.2 Pharmacokinetic profiles of marine bioactive peptides

Marine-derived neuroprotective peptides generally show low oral bioavailability due to enzymatic degradation in the gastrointestinal tract, poor membrane permeability, and large molecular size. Most peptides are hydrolyzed into small fragments before systemic absorption, and only a limited portion of intact peptides can enter the circulation (130).

Peptides with low molecular weight (< 3 kDa), high hydrophobicity, and specific amino acid compositions (e.g., Pro, Phe, Tyr, Trp) show better stability and absorption (131). A small number of cyclic peptides or lipophilic peptides are predicted to penetrate the BBB, whereas most linear peptides remain restricted by the BBB (109). These pharmacokinetic limitations directly affect the neuroprotective efficacy of marine peptides *in vivo*.

### 6.3 Chrononutrition, dosing patterns, and bioavailability of PUFAs

The cerebral bioavailability of PUFAs is strongly influenced by pharmacokinetic behavior and dosing strategies. Unlike bolus supplementation, sustained dietary DHA intake has been shown to maintain stable plasma DHA concentrations and enhance long-term brain absorption, reflecting the slow turnover rate of DHA in neural tissues (132). Maintaining stable plasma DHA availability is critical, as quantitative autoradiography and tracer studies have demonstrated that cerebral DHA is primarily derived from circulating unesterified DHA and LPC-DHA pools, rather than from postprandial spikes following acute bolus supplementation (129, 133). This finding is supported by human pharmacokinetic studies, which show that short-term or bolus dosing primarily increases triglyceride-bound DHA in circulation with limited immediate brain incorporation, whereas continuous dietary DHA intake gradually enriches plasma phospholipids and cerebrospinal fluid DHA levels (134). Due to the lack of sufficient pharmacokinetic data, optimal dosing regimens for marine peptides have not been established.

Diurnal fluctuations in circulating n-3 PUFAs may have functional implications for their tissue delivery, and disrupting this rhythm (e.g., by consuming n-3 PUFAs at night) could impact their efficacy (135). DHA has been shown to modulate both circadian rhythms and lipid metabolism (136). A study investigating 24-h fluctuations in plasma EPA and DHA concentrations in healthy individuals (during feeding and resting periods) collected plasma samples every 2 h from 22:00 to 22:00, with cosinor analysis used to quantify diurnal variation. Significant diurnal changes were observed in pooled plasma concentrations of both fatty acids, with DHA exhibiting the most prominent rhythmicity and a peak concentration at 17:43 (135). These findings underscore the potential importance of chrononutrition in optimizing DHA dosing strategies for maximal cerebral delivery and cognitive benefit.

## 7 Current challenges and synergistic application potential of marine-derived PUFAs, peptides, and supplements

### 7.1 Key challenges in clinical application of marine-derived PUFAs and peptides

Marine-derived PUFAs and bioactive peptides serve as critical regulators of neuronal integrity, synaptic plasticity and cognitive function. Nevertheless, their translational clinical application and further research advancement are severely restricted by several prominent challenges.

First, poor chemical stability, batch inconsistency and insufficient quality standardization represent major limitations for both marine-derived PUFAs and peptides. PUFAs are highly vulnerable to lipid peroxidation, and clinical evidence has verified a tight correlation among DHA dosage, chemical integrity and cognitive-improving efficacy. Impurities, oxidized byproducts and degraded components not only compromise bioactivity but also elicit adverse reactions including gastrointestinal discomfort. The standardization of extraction, purification, stabilization and encapsulation procedures remains unresolved, as process variability markedly reduces bioavailability and contributes to inconsistent outcomes across clinical trials (16). For marine-derived peptides, their structural stability is even more compromised. They are prone to enzymatic degradation during preparation, processing, storage and gastrointestinal digestion, which directly abolishes their neuroactive functions. Meanwhile, the lack of unified standards for peptide purity, molecular weight distribution and active sequence identification further hinders their reliable clinical translation (54).

Second, inefficient BBB penetration, unsatisfactory bioavailability and large individual differences constitute core bottlenecks. For PUFAs, although they can be incorporated into neuronal membranes, its passive transport into the CNS is inherently limited, representing a major obstacle for exerting neuroprotective effects. For marine peptides, their BBB permeability is generally poor due to relatively high molecular weight, hydrophilicity and rapid systemic degradation, greatly restricting direct CNS targeting. Furthermore, both PUFAs and peptides suffer from highly variable bioavailability, and personalized responses further complicate supplementation strategies. Individual variables such as sex, genetic background, baseline nutritional status and metabolic phenotypes significantly modify the clinical efficacy of supplementation (16, 137).

Third, the lack of standardized long-term clinical evaluations limits high-level evidence-based applications. Most preclinical and clinical studies focusing on PUFAs are limited to 12 months, providing insufficient data regarding their long-term effects on cognitive maintenance, age-related cognitive decline and the progression of neurodegenerative diseases. High-quality, long-term, large-scale randomized controlled trials for marine peptides are still lacking. Uniform dosing regimens, precise nutritional monitoring and standardized cognitive functional endpoints are also indispensable to establish evidence-based clinical recommendations for both marine PUFAs and peptides (47, 58, 82).

Last, safety evaluation represents a critical prerequisite for the clinical translation and large-scale application of marine-derived PUFAs and bioactive peptides. Marine n-3 PUFAs are highly unsaturated and extremely susceptible to oxidation under heat, light, oxygen, and moisture during processing, transportation, and storage. The peroxidation of PUFAs has emerged as a key driver of oxidative damage to cellular membranes leading to cell death (138). A significant increase in DHA-phospholipid hydroperoxides and products of phospholipid peroxidation was observed in aged memory impaired mice compared with aged memory unimpaired mice (139). Then, marine raw materials are facing increasingly severe pollution challenges, leading to potential accumulation of hazardous substances in PUFAs and peptide products, such as heavy metals, microplastics, and persistent organic pollutants (140, 141). Most bioactive peptides are prepared from fish, shellfish, shrimp, and other marine proteins, which carry inherent allergenic risks. Although enzymatic hydrolysis can reduce the allergenicity by breaking the intact epitope structure, some digestion-resistant immunodominant peptide fragments still retain the ability to activate mast cells and basophils, triggering allergic symptoms such as skin itching, rash, gastrointestinal discomfort, and even severe respiratory reactions or anaphylaxis in susceptible populations (142). In addition, a meta-analysis of 120,643 patients from 11 randomized clinical trials demonstrated that patients receiving high-dose purified EPA may incur additional bleeding risk, although its clinical significance is very modest (143). Another meta-analysis of 34 randomized clinical trials (n = 114,326) reported that high-doses of EPA/DHA (>1,500 mg/day) treatment was most likely to increase risk for atrial fibrillation in patients at high-risk for cardiovascular disease (144).

### 7.2 Synergistic effects of marine-derived PUFAs, peptides and supplements

Combinatorial interventions involving marine-derived PUFAs, bioactive peptides, and other neuroprotective supplements exert multimodal synergistic effects on cognitive function and brain health, as supported by consistent evidence from preclinical studies and clinical trials (145). Compared with single-component supplementation, combined regimens exhibit enhanced efficacy, reduced effective dosages, broader mechanistic coverage, and improved safety profiles, thereby representing a promising strategy for preventing and alleviating age-related cognitive impairment.

Marine-derived PUFAs and peptides confer enhanced neuroprotection through complementary biological mechanisms. In 15-month-old aging mice, combined administration of fish peptides and DHA/EPA improved memory performance, enhanced antioxidant activity, balanced gut microbiota composition, and attenuated neuroinflammation more effectively than either treatment alone (146). Similarly, the combination of DHA and walnut-derived hexapeptide (EPEVLR) incorporated into water-in-oil emulsions exerted greater cognitive improvement in D-galactose (D-gal) induced aging mice, accompanied by reduced oxidative stress, suppressed neuroinflammation, and stabilized cholinergic function (147). Mechanistically, peptides improved the stability and bioavailability of PUFAs, whereas PUFAs promoted synaptic targeting and neurotrophic signaling activated by peptides. Together, they coordinately upregulated BDNF/CREB signaling, reshaped microglial signature, and protected hippocampal structure and function (148–150).

Combinations of PUFAs with standardized herbal extracts or phytochemicals further strengthen cognitive outcomes. Supplements including *Bacopa monnieri*, *Ginkgo biloba*, and *Withania somnifera* extracts, as well as flavonoids and polyphenols, exert robust neuroprotective effects mainly through antioxidant, anti-inflammatory and neurotransmitter-modulating pathways. Notably, *Bacopa monnieri* and its main component, bacoside A, can promote hippocampal neurogenesis by increasing BDNF secretion, regulate neurotransmission, and modulate the expression of synaptic plasticity-related proteins, thereby improving spatial learning and memory (151). These characteristics enable them to act as ideal complementary agents with marine-derived PUFAs and peptides, allowing simultaneous targeting of multiple pathological pathways underlying cognitive decline. In an AlCl₃- and D-gal-induced mouse model of AD, combined DHA and *Ginkgo biloba* extract (EGb761) synergistically improved cognitive performance and preserved hippocampal morphology (89). Furthermore, long-term dietary intervention with EGCG, DHA, *α*-lipoic acid, and/or curcumin markedly reduced Aβ levels, suppressed microglial activation, and improved contextual memory in Tg2576 transgenic AD mice (152). At the cellular level, the triple combination of DHA, luteolin, and urolithin A (each at 5 μM) showed the strongest protective effects against Aβ-induced toxicity in human neuroblastoma BE(2)-M17 cells (153). Another study demonstrated that low dose combinations of curcumin (2.5 μM) with DHA or EPA (0.78 μM) synergistically suppressed LPS-induced prostaglandin E2 (PGE2), iNOS, and COX-2 expression (154).

Combinations of PUFAs with vitamins, minerals, and other lipid nutrients also improved cognitive performance in both preclinical models and human subjects. Co-administration of DHA and nervonic acid synergically enhanced spatial memory and fear memory function while remodeling brain fatty acid composition in normal mice (155). Oral administration of fish oil and choline for 30 days significantly upregulated key PUFA transporters including FATP1, FABP5 and Mfsd2a, thereby increasing mice brain DHA levels and enhancing learning and memory abilities (156). Similarly, combined fish oil, zinc, and selenium intervention for 7 weeks significantly improved learning and memory impairments in D-gal-induced aging mice, reduced A*β* levels in APP695-transfected CHO cells and inhibited β- and *γ*-secretase activities in PC_12_ cells (157). In clinical trials, combined fish oil (containing DHA/EPA) and multivitamin intervention resulted in modest cognitive benefits and synergistically improved cardiovascular biomarkers in healthy older adults (158). Daily oral intake of DHA and folic acid for 6 months exerted greater beneficial effects on cognitive function and reduced plasma inflammatory cytokines compared with either nutrient alone (159). A 24-month clinical trial further indicated that combined fish oil (DHA + EPA), carotenoids, and vitamin E significantly improved working memory in older adults in a dose-dependent manner (160).

Collectively, these findings demonstrate that combinatorial strategies integrating marine PUFAs, peptides, and other supplements represent a superior approach to supporting brain health and mitigating cognitive decline compared with single-component interventions. Studies investigating the combinatorial effects of these bioactive components on cognitive function are systematically summarized in Table 3.

**Table 3:** Clinical and preclinical evidence on combination of supplements with marine derived-PUFAs and peptides on cognitive function.

Type of experiments	Experimental model	Combination & dosage	Duration	Synergistic mechanism and key findings	Reference
In vitro cell model	BE(2)-M17 human neuroblastoma cells	DHA + luteolin + urolithin A (5 μM of each)	Acute cell assays (24 h pre-treatment + 72 h toxicity exposure)	Synergistic inhibition of Aβ1-42-induced toxicity; strongest cytoprotection in triple combination	(153)
	RAW 264.7 macrophage cells	Curcumin (2.5 μM) + DHA or EPA (0.78 μM)	24 h	Synergistic suppression of NO, PGE2, iNOS, COX-2, 5-LOX; induction of HO-1; enhanced anti-inflammatory activity at low doses	(154)
In vivo normal mice	6-week-old C57BL/6 normal mice	DHA (600 mg/kg) and nervonic acid (94 mg/kg)	6 weeks	Cognitive memory enhancement; brain fatty acids remodeling	(155)
	7-weeks and 15-month-old C57Bl/6 J normal mice	Fish peptides 2.9 mg/mouse + DHA/EPA 1.56 mg/mouse	11 weeks	Neuroprotection, and antioxidant activity; improved memory performance; gut microbiota rebalances	(146)
	8-month-old C57BL/6 J normal mice	Fish oil + choline (0.415 g/kg fish oil and 0.14 g/kg choline)	2 months	Upregulated Mfsd2a, FATP1, FABP5; enhanced BBB DHA transport (free & LPC form); increased acetylcholine synthesis; improved Morri’s water maze and passive avoidance performance	(156)
In vivo AD model mice	LPS-induced inflammation model mice	Fish peptides 5 mg/mouse + DHA 143 μg/mouse	18 days	Reduced microglial activation; increased neurotrophins	(150)
	AlCl_3_ and D-galactose (D-gal)-induced AD mouse model	DHA (200 mg/kg) + *Ginkgo biloba* extract (EGb761, 200 mg/kg)	8 weeks	Improved cognitive performance; preservation of hippocampal structure	(89)
	D-galactose-induced cognitive decline mice	DHA + walnut-derived hexapeptide (EPEVLR) in 2:1 molar water-in-oil emulsion	8 weeks	Greater cognitive improvement vs. monotherapy; reduced oxidative stress & neuroinflammation; stabilized cholinergic system; improved peptide stability via emulsion delivery	(147)
	D-gal aging mice; APP695-CHO cells; PC12 cells	Fish oil (EPA + DHA) + selenium + zinc (1 μmol/kg of each)	7 weeks	Improved Morris water maze performance; reduced Aβ1-40; decreased β- and *γ*-secretase activity; downregulated BACE1 and presenilin-1; APP processing inhibition	(157)
	Tg2576 transgenic AD mice	EGCG + DHA + α-lipoic acid (EDA) ± curcumin	12 months	Strongest reduction in amyloid plaque load; decreased Aβ40/Aβ42; reduced microglial activation; improved contextual memory	(152)
Clinical trial	Healthy older adults (age 50–70 years)	960 mg/day EPA + DHA + multivitamin	12 months	Modest improvements in selected cognitive domains; synergistic improvement of cardiovascular biomarkers	(158)
	Individuals with MCI (age ≥60)	DHA (800 mg/day) + folic acid (800 μg/day)	6 months	Improved cognitive performance; decreased plasma inflammatory cytokines	(159)
	Healthy older adults (age ≥65)	1 g fish oil (430 mg DHA + 90 mg EPA) + carotenoids (22 mg) + vitamin E (15 mg/day)	24 months	Significant improvement in working memory in a dose-dependent manner	(160)

## 8 Future research directions and development strategies

### 8.1 Standardization of processing and formulation methods

Supplement formulation and processing standardization are major barriers to the translational application of marine-derived PUFAs and bioactive peptides. For PUFAs, variations in extraction, purification, and encapsulation procedures lead to inconsistent DHA content, stability, and bioavailability, hindering inter-study reproducibility (16, 49). Future efforts should focus on unifying quality control criteria and standardizing key processes such as molecular distillation, microencapsulation, and the utilization of engineering PUFA synthase (16, 161, 162). For marine-derived peptides, standardization challenges extend to production (enzymatic hydrolysis/microbial fermentation parameters), purification (ultrafiltration, RP-HPLC protocols) and structural characterization (LC–MS/MS-based sequence verification). Unified standards for peptide purity, molecular weight distribution and active sequence identification are urgently needed to reduce batch variability. Overall, developing universal quality assessment systems and standardized workflows is critical to bridging preclinical research and clinical application.

### 8.2 Novel delivery systems for enhancing bioavailability and personalized nutrition strategies

Effective BBB transport is a critical prerequisite for marine-derived PUFAs to exert their neuroprotective effects. Novel delivery systems have emerged as a promising strategy to overcome the BBB penetration barrier. Among these delivery strategies, phospholipid-bound DHA/EPA has been demonstrated to yield superior cognitive outcomes and enhanced incorporation into neuronal membranes in both preclinical and human studies (129). Other innovative delivery modalities include DHA conjugates, liposomal carriers, nanoemulsions, exosome-based delivery, and polymeric nanoparticles (163). For marine-derived peptides, low oral bioavailability and BBB penetration are major challenges limiting their clinical translation (164). Recent studies have highlighted several encapsulation technologies to address these issues, including nanoemulsions, liposomal carriers, and biopolymer-based delivery systems. Encapsulation significantly improves peptide stability, enables controlled release, and preserves their biological activity, thereby enhancing their BBB penetration (165).

Personalized nutrition is key to maximizing the cognitive benefits of marine-derived PUFAs and peptides, as interindividual variability in response is influenced by genetic factors, baseline nutrient status, and metabolic phenotypes. For PUFAs, genetic differences such as APOE4 allele carrier status significantly impact outcomes. APOE4 carriers showed less pronounced cognitive improvements compared to non-carriers, highlighting the need for genotype-based dosing (166). Baseline DHA intake individuals with low habitual intake gain more cognitive benefits, while those with normal intake may only observe moderate effects (81). For marine peptides, bioavailability limitations (e.g., gastrointestinal degradation, poor BBB penetration) and individual differences further emphasize personalized strategies. Peptide bioavailability is strongly influenced by structural features, low-molecular-weight (<3 kDa) peptides with high hydrophobic amino acid content (e.g., Leu, Phe) exhibit better intestinal absorption and BBB penetration, but interindividual variations in digestive enzyme activity can alter hydrolysis and absorption efficiency (167). Therefore, precision nutrition strategies should integrate PUFAs and peptides based on individual profiles.

### 8.3 Long-term clinical trials for evidence-based recommendations

Despite well-documented preclinical neuroprotective effects of marine-derived PUFAs and peptides, evidence from long-term clinical trials remains limited, particularly for peptide-based interventions and their combinations with PUFAs. Most existing PUFA trials span 6–12 months, insufficient to capture sustained effects on cognitive decline or neurodegenerative disease progression, as DHA incorporation into neuronal membranes and peptide-mediated modulation of synaptic proteins occur gradually (168, 169). For peptides, clinical data are even scarcer, most studies focus on short-term (≤3 months) efficacy in healthy adults or animal models, with few investigations of long-term safety or durability of cognitive benefits (170). Therefore, long-term clinical trials (≥2–5 years) are urgently needed to validate the sustained efficacy and safety of marine bioactives.

## 9 Conclusion

Marine-derived bioactive peptides and PUFAs have emerged as promising nutritional and therapeutic interventions for mitigating age-related cognitive decline and neurodegenerative disorders. Core scientific findings of this review can be summarized as follows: First, long-chain n-3 PUFAs (especially DHA and EPA) and marine bioactive peptides exert memory-improving effects through complementary neuroprotective mechanisms. PUFAs maintain neuronal membrane integrity, suppress oxidative stress and neuroinflammation, regulate glial polarization, enhance synaptic plasticity, and modulate the microbiota–gut–brain axis. Peptides exert neuroprotection by inhibiting Aβ aggregation, reducing neuronal apoptosis, regulating cholinergic transmission, and activating neurotrophic signaling pathways. Second, phospholipid-bound DHA exhibits superior intestinal absorption, metabolic stability, and blood–brain barrier permeability compared with triglyceride-bound or free DHA, thereby supporting more effective cognitive improvement. Third, the combination of marine PUFAs, peptides, and other neuroprotective compounds produces synergistic effects that are superior to single-component supplementation.

However, several unresolved scientific questions and challenges remain. The precise mechanisms underlying BBB penetration of most marine peptides remain to be elucidated. The optimal molar ratio, dosage regimen, and long-term safety of combined PUFA–peptide interventions lack robust clinical validation. High-quality, long-term (≥2 years) clinical trials are still insufficient to confirm sustained cognitive benefits in aged or mild cognitive impairment populations. Standardized processes for production, quality control, and formulation of marine PUFAs and peptides have not been fully established. Advanced delivery systems that enhance stability, bioavailability, and brain targeting still require further development.

Overall, marine-derived PUFAs and bioactive peptides represent reliable and sustainable dietary resources for supporting brain health and delaying age-related cognitive decline. With further mechanistic research, standardized production, and long-term clinical evidence, their translation into practical nutritional and adjuvant therapeutic strategies will provide new avenues for the prevention and intervention of aging-related memory deficits.
